# Rare case of *Proteus mirabilis* native mitral valve endocarditis in an immunocompromised patient

**DOI:** 10.1186/s12879-021-06931-w

**Published:** 2021-12-14

**Authors:** Lindsay G. Grossman, Joseph M. Sharkey, David S. Grossman, Alan Hartman, Mina Makaryus, Kaushal B. Shah

**Affiliations:** 1 Perelman School of Medicine, University of Pennsylvania, 3400 Civic Center Blvd., Philadelphia, PA 19104 USA; 2 Northwell Health Partners in Critical Care, Manhasset, NY USA; 3 Plainview Hospital, Northwell Health, Plainview, NY USA; 4 Department of Cardiovascular and Thoracic Surgery, Northwell Health, Manhasset, NY USA; 5 Division of Pulmonary, Critical Care, and Sleep Medicine, Northwell Health, Manhasset, NY USA; 6 Division of Critical Care Medicine, South Shore University Hospital, Bay Shore, NY USA

**Keywords:** *Proteus mirabilis*, Infective endocarditis, Mitral valve, Immunocompromised, Case report

## Abstract

**Background:**

Bacterial infective endocarditis caused by *Proteus mirabilis* is rare and there are few cases in the literature. The natural history and treatment of this disease is not as clear but presumed to be associated with complicated urinary tract infection (cUTI).

**Case presentation:**

A 65-year-old female with a history of rheumatoid arthritis, factor V Leiden hypercoagulability, and prior saddle pulmonary embolism presented to the emergency department following a mechanical fall. Computed Tomography showed evidence of acute/subacute splenic emboli. Complicated UTI was likely secondary to a ureteral stone. Blood and urine cultures also grew out *P. mirabilis*. Transthoracic echocardiography revealed a mobile echogenic density on the anterior mitral valve (MV) leaflet consistent with a vegetation. The patient underwent MV replacement, and *P. mirabilis* was isolated from the surgically removed valve.

**Conclusions:**

We hypothesize that the patient’s immunocompromised status following steroid and Janus Kinase inhibitor usage for rheumatoid arthritis contributed to Gram-negative bacteremia following *P. mirabilis* UTI, ultimately seeding the native MV. Additional studies with larger numbers of *Proteus* endocarditis cases are needed to investigate an association between immunosuppression and *Proteus* species endocarditis.

## Background

Bacterial infective endocarditis (IE) is associated with increased morbidity and mortality and has become one of the leading life-threatening infection syndromes [1]. IE caused by *Proteus mirabilis* is exceedingly rare, and only a limited number of endocarditis due to *Proteus* spp. have been documented in the literature [2–4]. Due to this scarcity, there is a lack of definitive therapeutic guidelines of IE due to *P. mirabilis* [1, 2]. Documented treatment of *Proteus* endocarditis thus far has involved a prolonged course of antibiotics with up to half of patients requiring surgical intervention [5, 6]. Of the existing literature on IE due to *Proteus* spp., immunologic phenomena have not been frequently reported or well characterized [2]. Therefore, we present a unique case of mitral valve endocarditis caused by PM that highlights the potential role of immunosuppression in this uncommon presentation of IE.

## Case presentation

A 65-year-old female with a history of rheumatoid arthritis on chronic prednisone and scheduled Tofacitinib ER 11 mg q daily, factor V Leiden hypercoagulability, prior saddle pulmonary embolism, and no known valvular heart disease presented to the emergency department following a mechanical fall. On admission, she had a complaint of generalized fatigue. She had a temperature of 36.7 °C, heart rate (HR) of 97 beats per minute and a blood pressure (BP) of 155/86 with a normal lactate level. Her lungs were clear and there was no appreciable heart murmur noted. The remainder of the physical exam was normal.

**Fig. 1:**
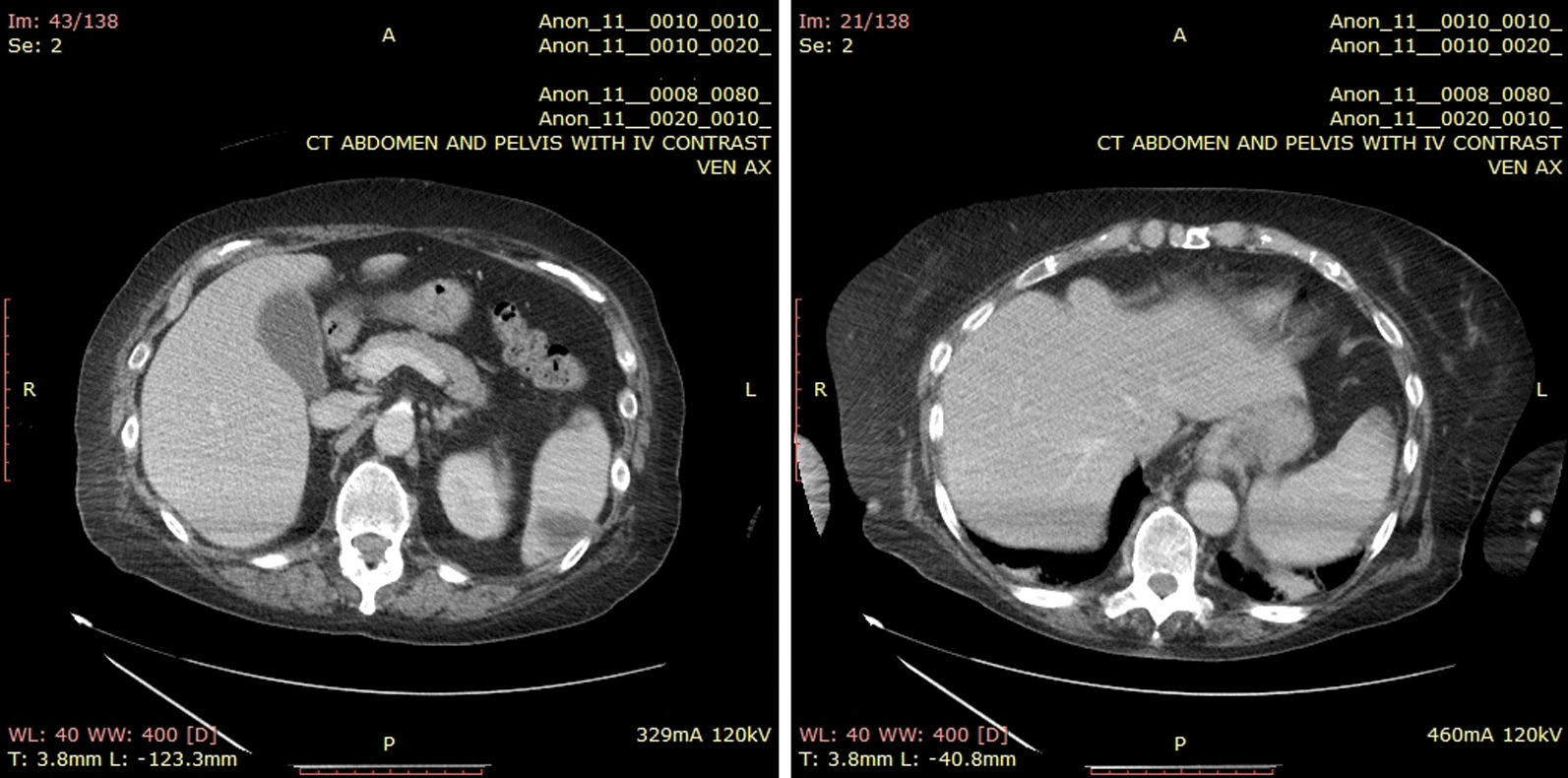
Abdominal/pelvic computed tomography scan showed evidence of acute/subacute splenic emboli

**Fig. 2:**
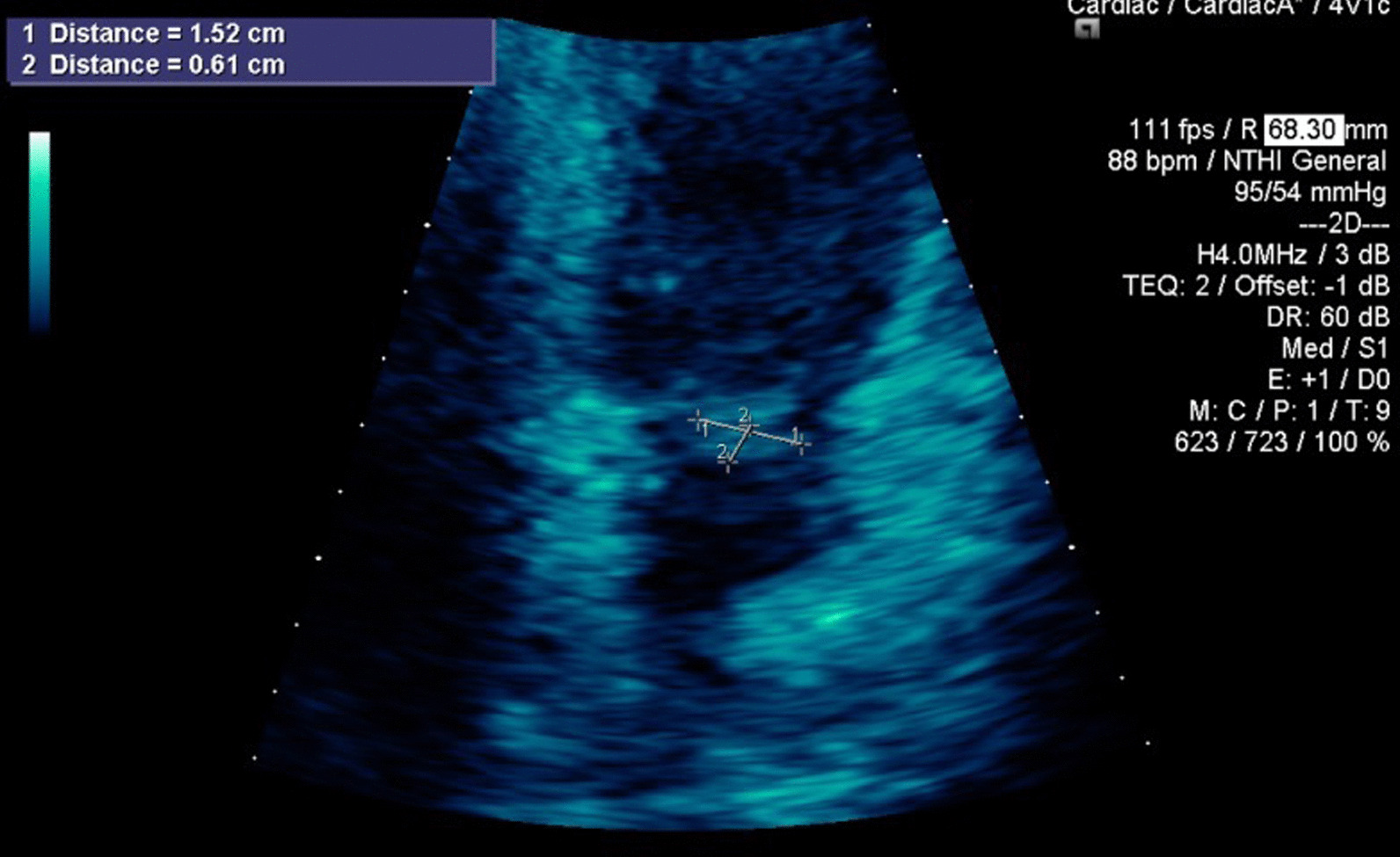
Transthoracic echocardiography revealed a 1.2 × 0.5 cm mobile echogenic density on the anterior leaflet of the mitral valve

**Fig. 3:**
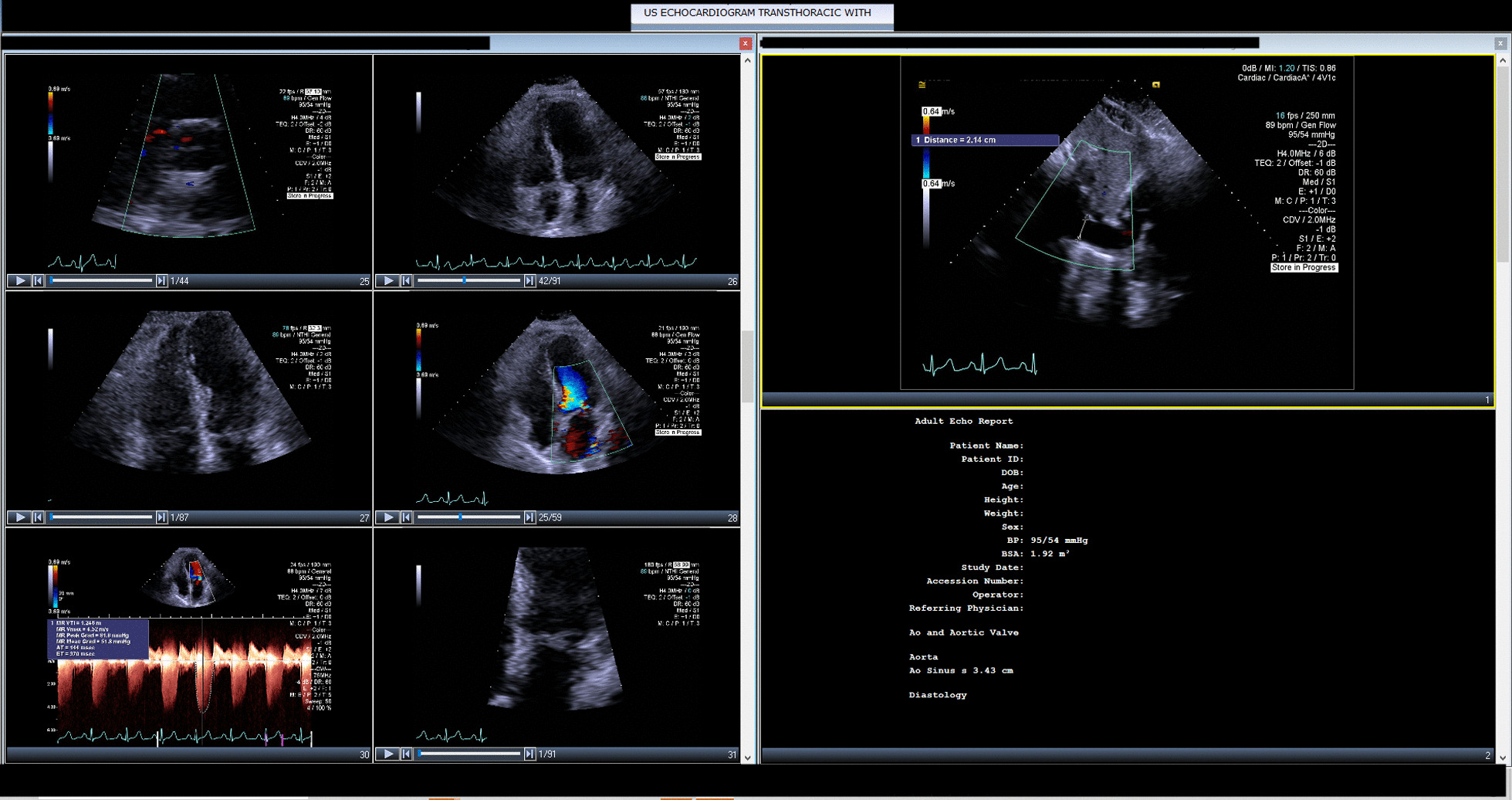
Transthoracic echocardiography revealed at least moderate mitral regurgitation

**Fig. 4:**
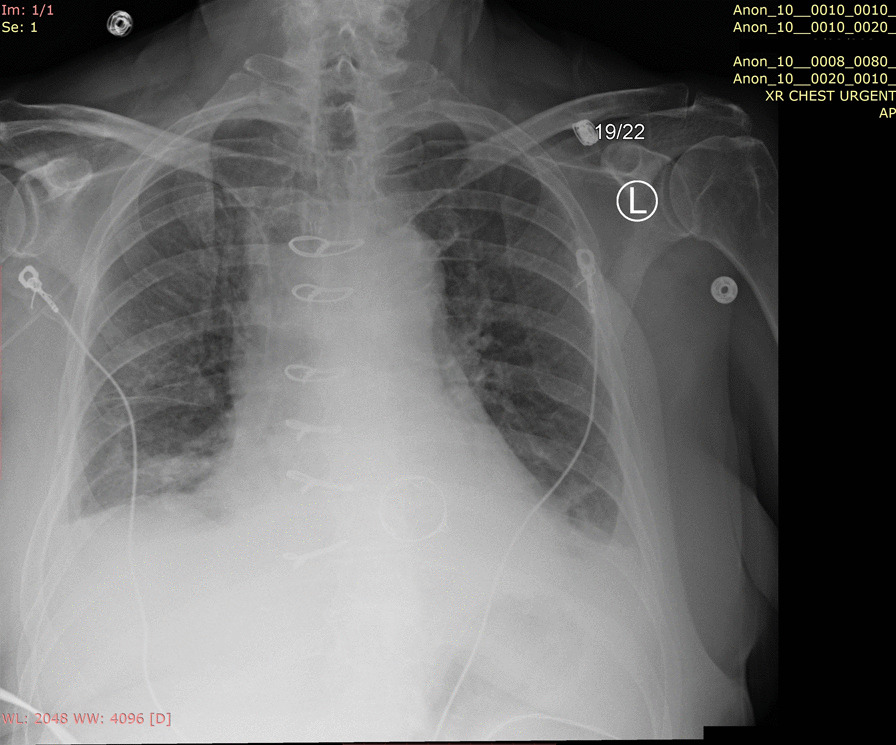
Chest X-ray revealed acute pulmonary edema

Her white blood cell count was 11,370/µL. Within 1 day of admission, patient had temperature of 40 °C, HR 104 bpm, BP dropping to 103/67 mmHg and white blood cell count increased to 18,480/µL. An abdominal/pelvic CT scan showed evidence of acute/subacute splenic emboli, with wedge-shaped zones of hypoattenuation in the inferior and superior aspects of the spleen (Fig. 1) and a 4 mm ureteral stone without hydronephrosis. Her urinalysis had bacteria, nitrite, and leukocyte esterase with only 5 WBCs. She was admitted to the general medical floor with a diagnosis of cUTI and was started on piperacillin/tazobactam 3.375 g every 8 h as an extended infusion. A transthoracic echocardiography revealed a 1.2 × 0.5 cm mobile echogenic density on the anterior leaflet of the MV (Fig. 2) with moderate mitral regurgitation (Fig. 3). Urine and blood cultures collected on the day of admission grew a pan sensitive strain of *P.* mirabilis (Table 1). Antibiotics were changed to ceftriaxone (2 g IV every 24 h) and gentamicin 5 mg/kg/day divided q 8 h. On hospital day 3, the patient developed respiratory distress and hypoxemia due to acute pulmonary edema on CXR (Fig. 4) requiring non-invasive positive pressure ventilation and was transferred to the intensive care unit. She became hypotensive and required intravenous nor-epinephrine. She developed new onset atrial fibrillation with a rapid ventricular response. Follow-up blood cultures continued to grow out *P. mirabilis.* Her white blood cell count peaked at 41,000 on hospital day 4. She subsequently required a second vasoactive agent, neosynephrine. Repeat echocardiography revealed an enlarging non-mobile vegetation 1.1 × 0.8 cm attached to the atrial side of the anterior MV leaflet. The mitral regurgitation was now severe with flow reversal into the pulmonary veins.

**Table 1:** Antimicrobial susceptibility test of the isolated *Proteus mirabilis*

	Urine clean catch		Blood culture	
	Sensitivity	Method (MIC)	Sensitivity	Method (MIC)
Amikacin	S	≤ 16	S	≤ 16
Amoxicillin/Clavulanic acid	S	≤ 8/4	N/A	
Ampicillin	S	≤ 8	S	≤ 8
Ampicillin/Sulbactam	S	≤ 4/2	S	≤ 4/2
Aztreonam	S	≤ 4	S	≤ 4
Cefazolin	S	≤ 2	S	≤ 2
Cefepime	S	≤ 2	S	≤ 2
Cefoxitin	S	≤ 8	S	≤ 8
Ceftriaxone	S	≤ 1	S	≤ 1
Ciprofloxacin	S	≤ 0.25	S	≤ 0.25
Ertapenem	S	≤ 0.5	S	≤ 0.5
Gentamicin	S	≤ 2	S	≤ 2
Levofloxacin	S	≤ 0.5	S	≤ 0.5
Meropenem	S	≤ 1	S	≤ 1
Nitrofurantoin	R	> 64	N/A	
Piperacillin/Tazobactam	S	≤ 8	S	≤ 8
Tobramycin	S	≤ 2	S	≤ 2
Trimethoprim/Sulfamethoxazole	S	≤ 0.5/9.5	S	≤ 0.5/9.5

*MIC* minimum inhibition concentration

**Fig. 5:**
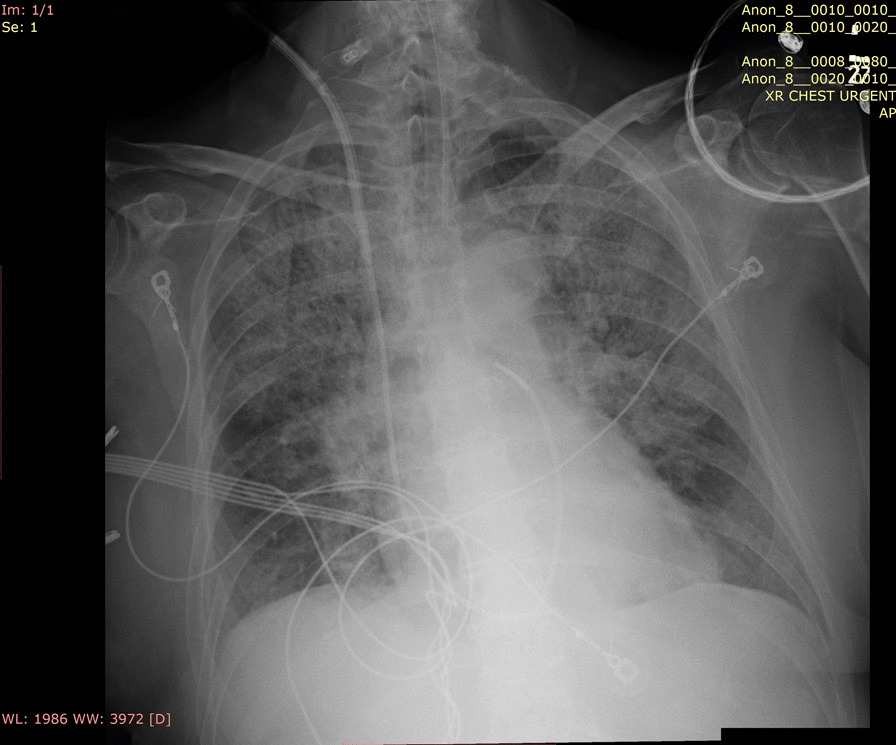
A pulmonary artery catheter was placed to guide management

The patient underwent cystoscopy for left ureteral stent placement for the noted stone. She continued to have mixed cardiogenic and septic shock secondary to *P. mirabilis*. bacteremia and MV endocarditis, now requiring continuous infusions of inotropic and two different vaso-active agents. A pulmonary artery catheter was placed to guide management (Fig. 5). On hospital day 7, she underwent surgical MV replacement with a 29 mm magnaease valve. Intra-op transesophageal echo revealed a well-seeded bioprosthetic mitral valve with no mitral regurgitation.

**Fig. 6:**
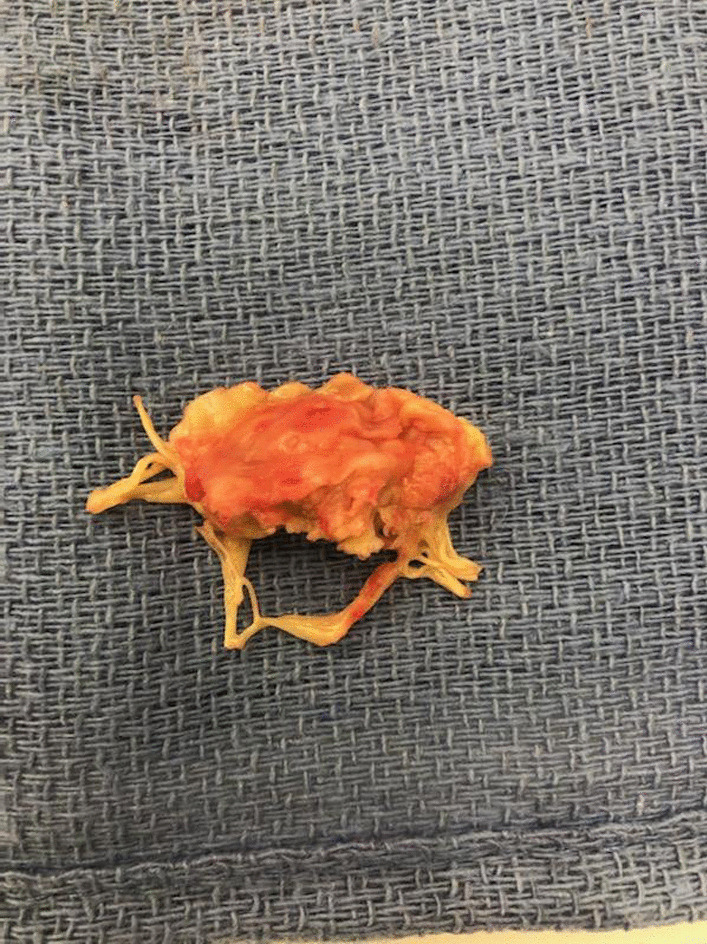
Pathologic specimen confirming acute mitral valve endocarditis

Repeat blood cultures negative for *P. mirabilis*. Continuous vasoactive agents and inotropes were tapered to off. Cardiac valve pathology confirmed acute MV endocarditis and intraoperative (Fig. 6). Ceftriaxone (2 g IV every 24 h) was given for 6 weeks post procedure and gentamicin was discontinued. The patient was discharged home post op day 5.

## Discussion and conclusions

*Proteus mirabilis* is Gram negative rod-shaped bacterium, member of the order *Enterobacteriales*, family *Enterobacteriaceae*, remains to be one of the common causes for complicated urinary tract infections consisting of flagellae and swarm cell differentiation contributing to its ability to cause ascending UTI and bacteremia. *P. mirabilis* is known to be producing urease, making urine alkaline from conversion of urea to ammonia and subsequently ammonium by consuming free hydrogen ion. Alkaline urine, in turn, facilitates struvite stones formation consisting of phosphate, carbonate and magnesium and subsequent vicious cycle of leukocytes, struvite, proteinaceous matrix & bacteria forms nidus for infection as infected staghorn calculus [7–11].

Cases of *P. mirabilis* endocarditis has rarely been reported in the current literature, with only 16 reports of IE caused by any *Proteus* species presented with high mortality of 43.8% [2]. In a large study of IE, only 3 out of 2761 (0.1%) definite cases were due to *Proteus* species [12]. While few cases have been documented, most patients with *Proteus* endocarditis presented with notably severe disease with high mortality [13–16]. The most recent incidence of native valve *Proteus* endocarditis was documented in 2016, in which the patient presented with *P. mirabilis* endocarditis of the aortic valve and was successfully treated with a dual antibiotic regimen of ceftriaxone and gentamicin [3]. Previously, two cases of *Proteus* endocarditis of native mitral valves were successfully treated without surgical intervention [17, 18].

Similar to factors found to be associated with non-HACEK Gram-negative bacillus (GNB) endocarditis, endocarditis due to enteric bacilli other than Salmonellae, and other previously reported cases of Proteus endocarditis, our patient presented with urinary tract infection (UTI) [3, 13, 19]. In a recent systematic review of *Proteus* endocarditis, 43.8% of patients had cocontaminant UTI [2]. Therefore, in retrospect, Proteus urosepsis was likely due to a 4 mm ureteral stone in our patient.

Interestingly, while embolic phenomena were frequently reported in cases of *Proteus* IE, only one other case reported splenic infarctions similarly seen in our patient [2, 18, 20]. Additionally, only 2 out of 14 (14.3%) documented cases of endocarditis due to any *Proteus* species reported immunologic involvement [2]. We hypothesize that our patient’s immunocompromised status following steroid and Janus Kinase inhibitor usage for rheumatoid arthritis contributed to Gram-negative bacteremia following *Proteus* urinary tract infection, ultimately seeding the native MV. Additional studies with larger numbers of *Proteus* endocarditis cases are needed to investigate an association between immunosuppression and *P. mirabilis* endocarditis. Overall, we summarize a case of *P. mirabilis* cUTI accentuating into persistent bacteremia and IE due to immunocompromised status of the host and yet being successfully managed by early surgical intervention and extended single antibiotic regimen.

## Data Availability

All data generated or analysed during this study are included within this published article.

## Abbreviations

IE: Infective endocarditis
cUTI: Complicated urinary tract infection
CT: Computed Tomography
TTE: Transthoracic echocardiography
MV: Mitral valve
GNB: Gram-negative bacteria
HR: Heart rate
BP: Blood pressure
